# Furnace Heat

**Published:** 1875-02

**Authors:** 


### FURNACE HEAT.

Probably there is not one person in a thousand, except such as are engaged in the manufacture or sale of some sort of heating devices, who will not declare that an old-fashioned, open wood fire-place, is the best heating arrangement ever used.

The reason for this almost universal preference is, unquestionably, because the air of an apartment thus heated is pleasant to breathe. The lungs are satisfied with it, and the occupant of the room feels well. Next to this, most people will assert, is the open grate for burning coal. This is pleasant to look upon, and, as in the case of the old-fashioned fire-place, the chimney or exit-place for the smoke and gases of combustion, also serves as a flue for the escape of the air of the room when consumed by the lungs. But it is not true that an open fire of wood or coal, or any other combustible, is necessarily a wholesome fire. It is not true that an apartment thus heated is a healthy one to occupy. This depends upon another condition, viz., the amount of fresh, pure air admitted. If you close your doors and windows tightly, seal them with weather strips, and take infinite pains to exclude the outer air, in order to save fuel, you cannot boast of a pure and wholesome atmosphere. Indeed, you cannot long boast of any air at all, because its "life" is certain to be exhausted by the lungs of the occupants, and by the demands of the fire on the hearth or in the grate. For you must remember that every iota of fuel or gas or oil consumed in your house, requires air and uses air, and leaves less for you and the other breathing animals. You cannot point to the good old days of blazing back-logs and big brass andirons, because the old time healthfulness argues nothing, since the houses in those days were full of chinks and crevices through which the eager air freely entered.

The whole philosophy of healthful heating, resolves itself into this inquiry—how shall we provide for our dwellings and places of business, pure air and warm air? It is not enough to solve merely the problem of supplying heat. Important as this is, it is insignificant as compared with the question of purity, newness, of the air which we must inhale. We *must not* be compelled to breathe over and over again the atmosphere of our houses. This we are all doing, day by day, simply because we know of no method of keeping warm except by shutting out the unused air, and getting up all the heat we can by the combustion of fuel.

It really seems reasonable after all, that the best way to heat an enclosure is to heat up good air outside, and then project it into the room in a volume large enough to supply warmth for the body, and fuel for the lungs. Our lungs can feed on warm air in winter, not less than in summer, if the air is new. What heating device, now in common use, better accomplishes the object than such a furnace as the "Gothic," made by Mr. A. M. Lesley, 224 West Twenty-third street, in this city? If there can be any better arrangement than this we should be glad to examine it, and will cheerfully commend it to our readers. For this question of how we shall heat our houses, is one of the most important known to us. We see disease and death prevailing in all directions on account of impure air. We see strong men dying suddenly, or suffering long because of the inhalations of the same old air, day after day, in offices heated by steam-coils. We seek to know just what is best for the masses, and are ever earnest in our commendation of the best when found.

